# Convex Versus Concave Emergence Profile of Implant‐Supported Crowns in the Aesthetic Zone: 3‐Year Results of a Randomized Controlled Trial

**DOI:** 10.1111/jcpe.70018

**Published:** 2025-08-20

**Authors:** Janina Endres, Franz J. Strauss, Marina Siegenthaler, Nadja Naenni, Ronald E. Jung, Daniel S. Thoma

**Affiliations:** ^1^ Clinic of Reconstructive Dentistry University of Zurich Zurich Switzerland; ^2^ Universidad Autonoma de Chile Santiago Chile; ^3^ Department of Periodontology, Research Institute for Periodontal Regeneration Yonsei University College of Dentistry Seoul Republic of Korea

**Keywords:** aesthetic zone, emergence profile, implant‐supported restoration, mucosal recession

## Abstract

**Aim:**

To evaluate the 3‐year clinical and radiographic outcomes of implant‐supported restorations with different emergence profiles (CONVEX vs. CONCAVE).

**Materials and Methods:**

A total of 47 patients received a single implant in the aesthetic zone and were allocated to one of three groups: (1) CONVEX: customized provisional with a convex emergence profile (*n* = 15); (2) CONCAVE: customized provisional with a concave profile (*n* = 16); (3) Control: no provisional restoration (*n* = 16). Final crowns in groups CONVEX and CONCAVE were fabricated to replicate the emergence profile of the respective provisional restorations. Follow‐ups were performed at baseline, 6 months, 1 year and 3 years. The primary outcome was mid‐facial mucosal recession and secondary outcomes included clinical, radiographic and aesthetic outcomes as well as profilometric measurements. Multivariable logistic regressions and mixed‐effects models were used to compare the groups.

**Results:**

Out of the 47 patients originally included, 42 were available for re‐examination at 3 years follow‐up. At 3 years, the frequency of mucosal recession amounted to 46.7% in group CONVEX, 13.3% in group CONCAVE and 40.0% in group Control. Adjusted logistic regression models revealed that the CONVEX group was significantly more likely to show recessions at 3 years (odds ratios [ORs]: 7.3, 95% CI: 1.02–52.14, *p* = 0.048) when compared with the CONCAVE group. No statistically significant difference in recession frequency was observed between the CONVEX and CONCAVE groups between the 1‐ and 3‐year follow‐ups (OR: 3.7, 95% CI: 0.30–46.09, *p* = 0.303).

**Conclusion:**

The emergence profile design significantly influences soft tissue stability predominantly within the first year after crown insertion. Whenever clinically feasible, a CONCAVE profile is preferable in the aesthetic zone to maintain the level of the mid‐facial mucosal margin and reduce the frequency of recessions.

**Trial Registration:** German Clinical Trials Register: DRKS00009420

## 1 Introduction

The benefits of using a provisional restoration after implant placement have been widely debated (De Bruyn et al. 2013; Jemt 1997). The design of the provisional restoration plays a crucial role in shaping the transition from the implant platform to the broader, oval‐shaped mucosal margin coronally. This design influences the peri‐implant mucosal healing and maturation and is also linked to early clinical outcomes, such as marginal bone levels (Souza et al. 2018; Strauss, Park, et al. 2024). While short‐term benefits, such as increased papilla height, have been observed, mid‐term outcomes do not result in significant differences compared to sites without provisional restorations (Jemt 1999). This aligns with findings that the most significant changes of the peri‐implant tissue architecture occur within the first year of loading (Donker et al. 2024; Small and Tarnow 2000).

Beyond the initial healing phase, the prosthetic design of the emergence profile has long‐term implications for aesthetics (Gomez‐Meda et al. 2021; Su et al. 2010), peri‐implant mucosal dimensions (Puisys et al. 2023; Rungtanakiat et al. 2024) and overall peri‐implant tissue health (Pelekos et al. 2023). Certain designs have been associated with complications including mucosal recession (Rompen et al. 2007; Siegenthaler et al. 2022), mucositis (Rungtanakiat et al. 2024) and peri‐implantitis (Katafuchi et al. 2018; Misch et al. 2025). As a result, achieving a biologically stable emergence profile has become a key focus in both research and clinical practice.

Several clinical techniques have been proposed to condition the emergence profile using provisional restorations, including: (1) the cervical contouring concept (Bichacho and Landsberg 1997), (2) the dynamic compression technique (Wittneben et al. 2013) and (3) the selective pressure method (Nam and Aranyarachkul 2015). These methods vary in complexity and shape of the resulting emergence profile contour. Most studies describe a CONCAVE emergence profile as the preferred design (De Rouck et al. 2008; Gonzalez‐Martin et al. 2020; Nam and Aranyarachkul 2015; Rompen et al. 2007). However, a CONVEX emergence profile is often recommended when an implant is placed too palatally or lingually (Chu 2020; Esquivel et al. 2021; Steigmann et al. 2014) or in the upper part of the transmucosal zone near the marginal mucosa (Seyssens et al. 2020). Despite these recommendations, emergence profiles are often chosen arbitrarily or delegated to the dental technician, as limited research has systematically investigated their impact on clinical and aesthetic outcomes, particularly in prosthetically ideal implant positions.

The terms ‘CONVEX’ and ‘CONCAVE’ have traditionally been used to describe the shape of implant‐supported crowns, gaining attention after a randomized controlled trial reported a higher frequency of mid‐facial recessions with CONVEX crowns (Siegenthaler et al. 2022). However, whether these aesthetic complications worsen over time or remain stable, along with their corresponding clinical outcomes, remains unclear. The aim of the present study was, therefore, to assess 3‐year clinical, radiographic and profilometric outcomes of implant‐supported restorations with a differing emergence profile.

## 2 Material and Methods

### 2.1 Study Design and Patient Population

This study presents a 3‐year follow‐up of an randomized controlled trial (RCT) with three parallel groups and was approved by the local ethics committee (KEK‐Nr 2015‐0284 No. 2012‐0147). A total of 47 patients were consecutively enrolled and received a single dental implant (OsseoSpeed Tx, Astra Tech Implant System, Dentsply Sirona Implants) in the aesthetic zone of the maxilla or mandible (incisors, canines or premolars) following a prosthetically driven approach. Further details of the study design have been previously reported (Siegenthaler et al. 2022).

### 2.2 Randomization and Group Allocation

Three to four months after implant placement, patients were randomly assigned to one of three groups. The allocation was based on a computer‐generated randomization list. The groups were as follows:

1. *CONVEX*: customized provisional screw‐retained crown with a CONVEX contour (*n* = 15).
2. *CONCAVE*: customized provisional screw‐retained crown with a CONCAVE contour (*n* = 16).
3. *Control*: No provisional restoration was applied and a standardized healing abutment was used (*n* = 16).

### 2.3 Clinical and Laboratory Procedures

For patients in the CONVEX and CONCAVE groups, impressions were taken and temporary crowns with an initially under‐contoured design were fabricated using a temporary titanium abutment (Temp Abutment TX, Astra Tech Implant System, Dentsply Sirona Implants). The provisional crowns were then modified based on the assigned group:

- The under‐contoured shape was gradually adjusted by applying a thin layer of flowable composite material to achieve either a CONVEX or CONCAVE emergence profile.
- The emergence profile was shaped starting 1–2 mm above the abutment's neck.

**FIGURE 1:**
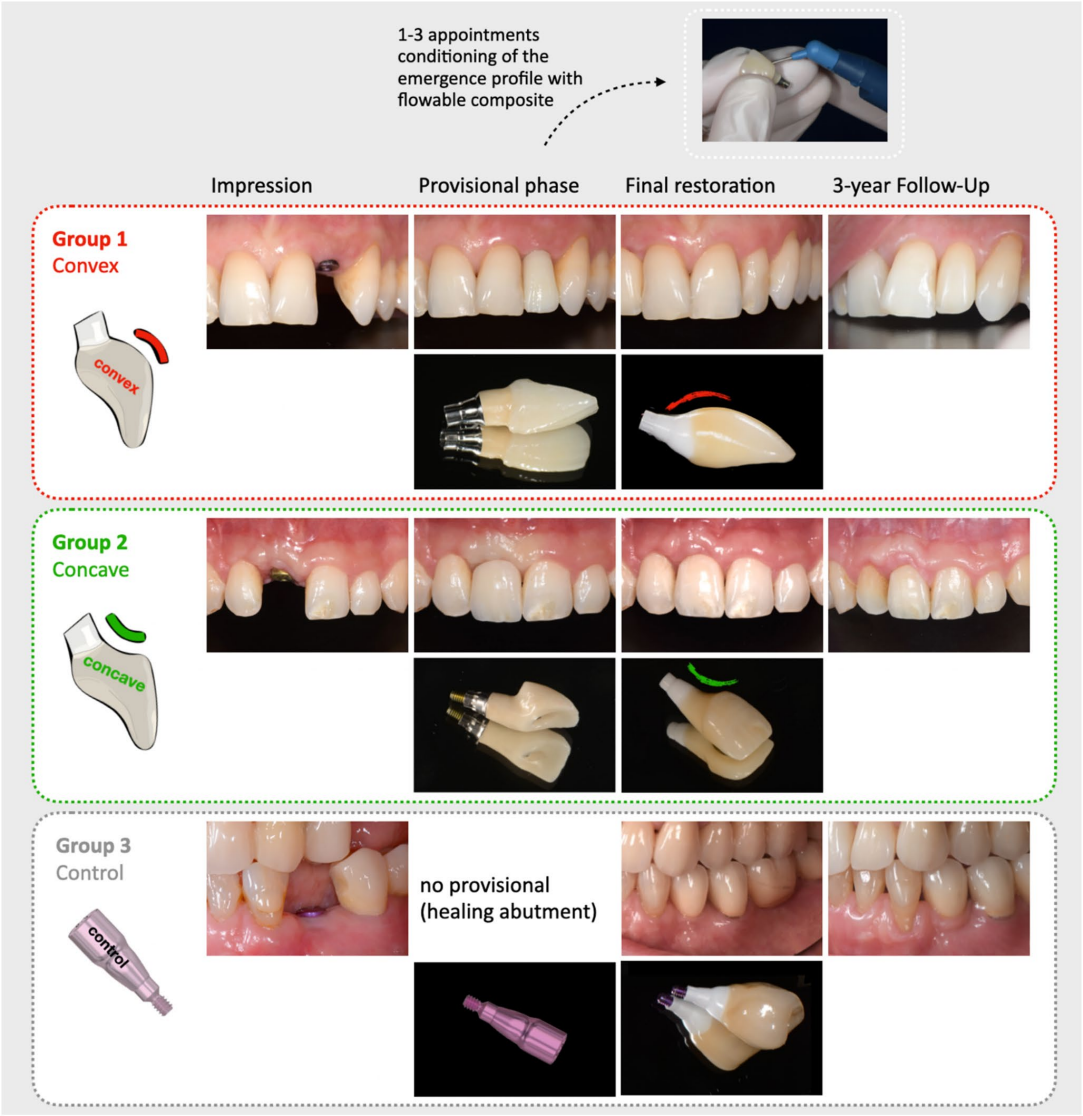
Study workflow.

For the Control group, a titanium healing abutment (HealDesign EV or Healing Uni EV, Astra Tech Implant System) was inserted (Figure 1). The abutment diameter was selected based on the individual anatomical conditions of the implant site. All Control crowns were fabricated following a straight and flat emergence profile.

A final implant impression was taken to fabricate the screw‐retained monolithic zirconia crowns, which were then cemented onto individualized zirconia abutments (Atlantis, Dentsply Sirona Implants), replicating the emergence profile of the provisional restorations (Figure 1). The study timeline is summarized in Figure S1.

### 2.4 Maintenance and Follow‐Up

Patients underwent a baseline examination 7–10 days after the final restoration insertion. Subsequent follow‐up visits were scheduled at 12 and 36 months. A blinded and calibrated examiner, who was not involved in the surgical or prosthetic procedures, conducted all follow‐up examinations. The following outcomes were assessed:

#### 2.4.1 Mid‐Facial Mucosal Recession

Mid‐facial mucosal recession was defined as an apical shift of the mid‐facial mucosa at the implant‐supported crown compared to baseline (crown insertion). The extent of recession (mm) was determined by measuring the difference in clinical crown length between baseline and follow‐ups using a periodontal probe. For the primary analysis, the mid‐facial mucosal recession was dichotomized into recession (0 > mm apical shift) or absence of recession (0 mm shift or coronal shift).

#### 2.4.2 Aesthetic and Clinical Outcomes

The modified Pink Aesthetic Score (PES) was evaluated (Belser et al. 2009). Probing depth (PD), bleeding on probing (BOP) and plaque were assessed at 6 sites. Buccal keratinized tissue (KT) was measured using a periodontal probe. Buccal soft tissue thickness was assessed using an ISO 15 endodontic file, inserted 1 mm below the mucosal margin at the central aspect of the implant crown.

#### 2.4.3 Linear and Profilometric Outcomes

Contour changes were assessed by a blinded examiner using digital analysis software (SMOP, Swissmeda, Switzerland) as previously described (Siegenthaler et al. 2022; Strauss, Fukuba, et al. 2024; Strauss et al. 2022; Thoma et al. 2024) at 1 and 3 mm apical to the mid‐facial mucosal margin (Figure S2) and two regions of interest (ROI).

#### 2.4.4 Marginal Bone Levels

Standardized single‐tooth radiographs were taken at baseline and each follow‐up. Marginal bone levels (MBLs) were assessed using ImageJ software (National Institute of Health, Bethesda, MD) as previously described (Siegenthaler et al. 2022).

#### 2.4.5 Diagnosis of Peri‐Implant Conditions

Peri‐implantitis was diagnosed according to the 2017 World Workshop (Berglundh et al. 2018). Peri‐implant mucositis was defined according to the updated ID‐COSM consensus (Tonetti et al. 2023) as bleeding (more than one spot on gentle probing) without bone loss beyond initial crestal bone remodelling (Herrera et al. 2023).

### 2.5 Sample Size Calculation

The sample size was determined using G*Power software (Faul et al. 2007), based on Fisher's exact test for independent proportions (two‐sided), with a 5% α level and 80% statistical power. A 56% difference in mucosal recession frequency was considered clinically relevant, based on reported mid‐facial recession rates ranging from 7% (Raes et al. 2011) to 64% (Cordaro et al. 2009; Cosyn et al. 2012) in immediate implant placement. To account for a 20% dropout rate, 47 patients were enrolled.

### 2.6 Statistical Analysis

Descriptive statistics were calculated, including means, standard deviations (SD) and medians for metric parameters, as well as frequencies for categorical parameters. To predict the presence of mid‐facial mucosal recession (yes/no) at the 3‐year follow‐up, a multivariable logistic regression analysis was performed, adjusting for soft tissue thickness and keratinized tissue width. Changes in clinical, aesthetic, profilometric and radiographic outcomes within and between treatment groups were assessed using linear mixed‐effects models, accounting for the within‐subject correlation of repeated measurements. Fixed factors included treatment group, time and their interaction, allowing estimation of treatment effects at each time point, while patients were considered random factors. Differences between groups were estimated using a linear contrast command. Model assumptions were visually inspected through residual plots, including Q–Q plots and histograms. To evaluate the reliability of the outcome assessor for PES and profilometric measurements, the two‐way mixed, single rater and absolute agreement intra‐class correlation coefficient (ICC) was used. The data were analysed using the per‐protocol approach. In addition, to evaluate the robustness of the primary outcome, the data were also evaluated applying an intention‐to‐treat (ITT) approach and imputing missing data using the last observation carried forward method (Newgard and Lewis 2015). The significance level (α) was set at 5%. Statistical analyses were conducted using Stata v18.0 (StataCorp).

## 3 Results

### 3.1 Patients

**FIGURE 2:**
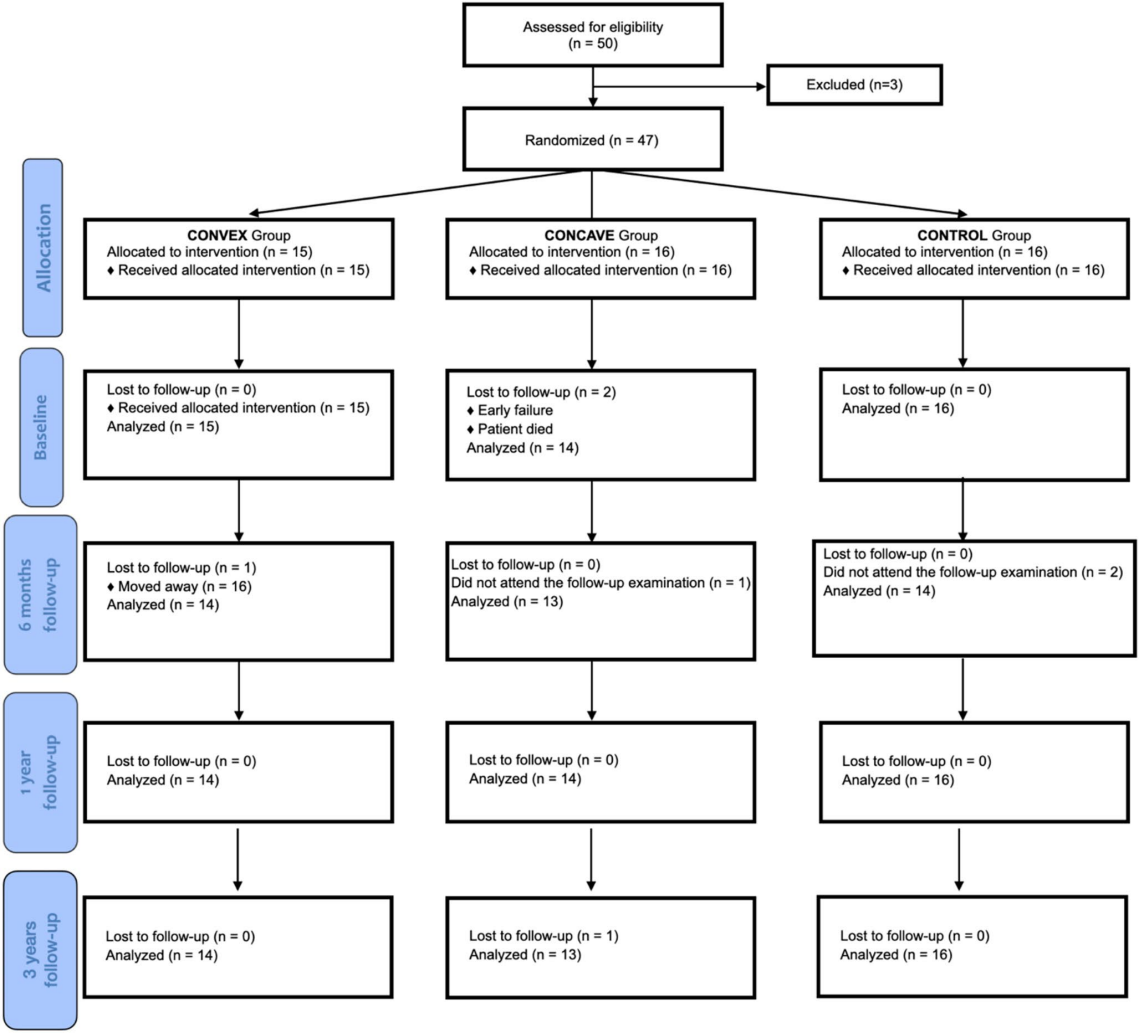
Consort diagram.

Detailed demographic data are provided in Table S1, and implant locations are illustrated in Figure S3. At the 3‐year follow‐up, 43 patients remained in the study. The study flow is depicted in the CONSORT diagram (Figure 2). The survival rates for both implants and restorations remained at 100% from crown insertion throughout the 3‐year follow‐up.

### 3.2 Mid‐Facial Mucosal Recession (Primary Outcome)

One year after implant‐supported crown insertion, the frequency of mucosal recession was 64.3% in the CONVEX group, 14.3% in the CONCAVE group and 31.4% in the Control group. At the 3‐year follow‐up, the frequency was 46.7% in the CONVEX group; it remained low at 13.3% in the CONCAVE group and was 40.0% in the Control group.

Adjusted logistic regression models (Table 1) revealed that the CONVEX group was significantly more likely to develop mucosal recession at both 1‐ and 3‐year follow‐ups compared to the CONCAVE group (reference group), with odds ratios (ORs) of 13.3 (95% confidence interval [CI]: 1.2–138.5, *p* = 0.003) and 7.3 (95% CI: 1.0–52.1, *p* = 0.048), respectively. No significant association was found between the Control group and the occurrence of mucosal recession at the 1 year follow‐up (OR: 3.3, 95% CI: 0.2–37.7, *p* = 0.335) nor at the 3 years follow‐up (OR: 3.1, 95% CI: 0.4–20.3, *p* = 0.232). Similarly, the ITT analysis using the last observation carried forward method showed a clear trend towards greater mucosal recession in the CONVEX group compared to the CONCAVE group at the 3‐year follow‐up (OR: 4.8, 95% CI: 0.8–27.4, *p* = 0.072) (Table S2). Additional analyses, whether unadjusted for baseline soft tissue covariates (Table S3), adjusted for soft tissue thickness (Table S4) or adjusted for keratinized mucosa (Table S5) yielded similar results.

**TABLE 1:** Multivariable logistic regression analysis for predicting the presence of recessions (yes/no) adjusted for treatment, soft tissue thickness and keratinized tissue width.

	1 year follow‐up			3 years follow‐up		
	OR	95% CI	*p*	OR	95% CI	*p*
Treatment						
CONCAVE (reference)	1	1	1	1		
CONVEX	13.3	1.2–138.5	0.030*	7.3	1.0–52.1	0.048*
CONTROL	3.3	0.29–37.74	0.335	3.1	0.5–20.3	0.232
Soft tissue thickness (mm)	0.9	0.59–1.52	0.851	1.3	0.8–2.0	0.236
Keratinized tissue width (mm)	0.8	0.45–1.65	0.657	0.9	0.5–1.5	0.575

*Note*: Odds‐ratio, 95% confidence interval, and *p*‐value obtained using multivariable logistic regression adjusted for soft tissue thickness and keratinized width based on complete case analysis. * Bold values indicate statistically significant differences.

Abbreviations: CI, confidence interval; OR, odds‐ratio.

No statistically significant difference between the CONVEX and CONCAVE groups was found for the interval between the 1‐ and 3‐year follow‐ups (OR: 3.7, 95% CI: 0.3–46.0, *p* = 0.303; Table S6).

### 3.3 Magnitude of Recession

At the 1‐year follow‐up, the mean mucosal recession was 0.72 ± 0.60 mm (median: 0.50 mm) in the CONVEX group, 1.00 ± 0.00 mm (median: 1.00 mm) in the CONCAVE group and 0.90 ± 0.65 mm (median: 0.50 mm) in the Control group. By the 3‐year follow‐up, the mean recession amounted to 0.15 ± 1.10 mm (median: 0.25 mm) in the CONVEX group. Conversely, in both the CONCAVE group (−0.69 ± 1.43 mm; median: 0.00 mm) and Control group (−0.13 ± 1.06 mm; median: 0.00 mm) mean values were negative, indicating a coronal shift of the mid‐facial mucosal margin.

### 3.4 Clinical Outcomes

At 3 years follow‐up, there were no significant differences in PD values between the groups (Table 2), with median values of 3.1 mm in the CONVEX group, 2.9 mm in the CONCAVE group and 2.8 mm in the Control group (intergroup *p* = 0.775; Table 2). Similarly, BOP values showed no significant differences, with median values of 0.3 in the CONVEX group, 0.1 in the CONCAVE group and 0.3 in the Control group. Plaque levels also did not differ between the groups, with a median score of 0 across all three groups (Table 2). The prevalence of peri‐implant mucositis was 53.8% in the CONVEX group, 33.3% in the CONCAVE group and 69.0% in the Control group, with no significant difference between the groups (intergroup *p* = 0.235). As for peri‐implantitis, only one patient in the CONCAVE group presented peri‐implantitis, whereas no case of peri‐implantitis was diagnosed in the CONVEX or Control groups.

**TABLE 2:** Clinical parameters within each group at 3 years of follow‐up.

Group	CONVEX		CONCAVE		CONTROL		Intergroup *p*
	Mean (SD)	Median (Q1, Q3)	Mean (SD)	Median (Q1, Q3)	Mean (SD)	Median (Q1, Q3)	
PD	3.2 (0.8)	3.1 (2.6, 3.5)	3.0 (0.5)	2.9 (2.6, 3.3)	3.1 (0.8)	2.8 (2.6, 3.8)	0.775
BOP	0.3 (0.2)	0.3 (0.1, 0.5)	0.2 (0.1)	0.1 (0.0, 0.3)	0.3 (0.2)	0.3 (0.1, 0.5)	0.208
PI	0.1 (0.2)	0 (0, 0.1)	0.0 (0.0)	0 (0, 0)	0.0 (0.1)	0 (0, 0)	0.165

*Note*: Intergroup differences were tested using Kruskal–Wallis test.

Abbreviations: BOP, bleeding on probing; PD, probing depth; PI, Plaque index.

### 3.5 Aesthetic Outcomes

#### 3.5.1 Pink Aesthetic Score

PES values at all time points showed no significant differences between the groups (*p* = 0.735) (Table 3). No significant interaction between group and time was observed (group#time, *p* = 0.932).

**TABLE 3:** PES values and frequency distribution per group at 3 years follow‐up.

Group	CONCAVE (*n* = 13)	CONVEX (*n* = 14)	CONTROL (*n* = 15)
Mesial papilla			
0	1 (8%)	3 (21%)	1 (7%)
1	8 (61%)	5 (36%)	7 (47%)
2	4 (31%)	6 (43%)	7 (47%)
Distal papilla			
0	1 (8%)	1 (7%)	2 (13%)
1	10 (77%)	8 (57%)	9 (60%)
2	2 (15%)	5 (36%)	4 (27%)
Curvature of facial mucosa			
0	0 (0%)	1 (7%)	0 (0%)
1	3 (23%)	3 (21%)	8 (53%)
2	10 (77%)	10 (71%)	7 (47%)
Level of facial mucosa			
0	0 (0%)	1 (7%)	1 (7%)
1	1 (8%)	6 (43%)	5 (33%)
2	12 (92%)	7 (50%)	9 (60%)
Root convexity/soft tissue colour and texture			
0	1 (8%)	0 (0%)	1 (7%)
1	11 (84%)	8 (57%)	11 (73%)
2	1 (8%)	6 (43%)	3 (20%)

### 3.6 Linear and Profilometric Outcomes

#### 3.6.1 Reliability of Measurements

The ICC for the linear measurements at 1 and 3 mm below the mid‐facial mucosal margin amounted to 0.95 [95% CI, 0.78; 0.99] and 0.96 [95% CI, 0.87; 0.99], respectively. For ROI‐1, the ICC amounted to 0.96 [95% CI, 0.87; 0.99] and for ROI‐2, it amounted to 0.98 [95% CI, 0.86; 0.99].

#### 3.6.2 Linear Measurements

There were no significant differences in peri‐implant tissue width between the groups at 1 mm and 3 mm apical to the mid‐facial mucosa (Table 4). Additionally, no significant group‐by‐time interaction (group#time) was observed (*p* > 0.05).

**TABLE 4:** Profilometric linear changes within each group across the different timepoints.

Profilometric									
Group	CONVEX		CONCAVE		CONTROL		*p* (angle effect)	*p* (time effect)	*p* (angle#time interaction)
Timepoint	Mean (SD)	Median (Q1, Q3)	Mean (SD)	Median (Q1, Q3)	Mean (SD)	Median (Q1, Q3)			
1 mm									
ΔBL—6 months	0.0 (0.5)	−0.1 (−0.2, 0.2)	−0.1 (0.22)	−0.2 (−0.3, −0.0)	−0.1 (0.39)	−0.1 (−0.3, 0.2)	0.346	0.06	0.826
ΔBL—1‐year	−0.2 (0.4)	−0.2 (−0.5, 0.0)	−0.3 (0.25)	−0.3 (−0.4, −0.0)	−0.3 (0.25)	−0.3 (−0.4, −0.1)			
ΔBL—3‐year	−0.2 (0.3)	−0.2 (−0.4, −0.0)	−0.4 (0.37)	−0.4 (−0.6, −0.1)	−0.4 (0.45)	−0.4 (−0.6, −0.1)			
3 mm									
ΔBL—6 month	0.0 (0.4)	−0.1 (−0.3, 0.1)	−0.3 (0.4)	−0.2 (−0.3, −0.1)	−0.2 (0.6)	−0.1 (−0.4, 0.1)	0.479	0.820	0.396
ΔBL—1‐year	−0.2 (0.4)	−0.1 (−0.3, −0.0)	−0.4 (0.5)	−0.3 (−0.8, −0.1)	−0.4 (0.5)	−0.4 (−0.8, −0.1)			
ΔBL—3‐year	−0.2 (0.3)	−0.2 (−0.4, 0.2)	−0.6 (0.5)	−0.6 (−0.8, −0.2)	−0.6 (0.7)	−0.5 (−0.6, −0.1)			

*Note*: Differences were tested using a linear mixed‐effects model with treatment (shape), time and their interaction; *p*‐values are indicated. Wald's Chi‐squared statistic was used to conclude about main effects and interactions between treatment and time.

Abbreviations: Q1, first quartile; Q3, third quartile; SD, standard deviation.

#### 3.6.3 Profilometric Measurements

At region of interest 1 (ROI‐1), profilometric contour changes did not differ significantly among the three groups (*p* = 0.346), although all groups showed a trend towards slight loss of contour over time (*p* = 0.105) (Table 5). No significant group‐by‐time interaction (group#time) was observed (*p* = 0.826) (Table 5).

**TABLE 5:** Profilometric contour changes within each group across regions of interest (ROI) and time points.

Group	CONVEX		CONCAVE		CONTROL		*p* (shape effect)	*p* (time effect)	*p* (shape#time interaction)
Timepoint	Mean (SD)	Median (Q1, Q3)	Mean (SD)	Median (Q1, Q3)	Mean (SD)	Median (Q1, Q3)			
ROI 1									
ΔBL—6 months	0.1 (0.5)	−0.1 (−0.2, 0.2)	−0.1 (0.2)	−0.1 (−0.2, −0.1)	−0.1 (0.4)	0.0 (−0.2, 0.1)	0.482	0.105	0.431
ΔBL—1‐year	−0.2 (0.3)	−0.1 (−0.3, −0.0)	−0.2 (0.2)	−0.2 (−0.3, −0.0)	−0.2 (0.2)	−0.2 (−0.3, −0.1)			
ΔBL—3‐year	−0.1 (0.3)	−0.1 (−0.3, 0.0)	−0.1 (0.5)	−0.2 (−0.4, −0.0)	−0.3 (0.4)	−0.3 (−0.6, −0.1)			
ROI 3									
ΔBL—6 months	0.2 (0.5)	0.0 (−0.0, 0.3)	−0.2 (0.3)	−0.2 (−0.3, −0.1)	−0.1 (0.5)	−0.1 (−0.3, 0.1)	**0.038**	0.081	0.148
ΔBL—1‐year	−0.1 (0.4)	−0.0 (−0.1, 0.1)	−0.3 (0.4)	−0.2 (−0.5, 0.0)	−0.3 (0.4)	−0.3 (−0.7, 0.0)			
ΔBL—3‐year	0.1 (0.3)	−0.0 (−0.1, 0.2)	−0.4 (0.4)	−0.3 (−0.6, −0.1)	−0.4 (0.5)	−0.3 (−0.5, −0.1)			

*Note*: Differences were tested using a linear mixed‐effects model with treatment (shape), time and their interaction; *p*‐values are indicated. Wald's Chi‐squared statistic was used to conclude about main effects and interactions between treatment and time.

Abbreviations: Q1, first quartile; Q3, third quartile; SD, standard deviation.

At ROI‐3, a significant difference between the groups was observed (*p* = 0.038) (Table 5) but the magnitude of the change was not significantly different among the groups. No group‐by‐time interaction (group#time) was observed (*p* = 0.148) (Table 5).

### 3.7 Marginal Bone Levels

MBL values at all time points showed no significant differences between the groups (*p* = 0.961) (Table S6). Additionally, there were no significant bone changes over time (*p* = 0.805). No significant interaction between group and time was found (group#time, *p* = 0.301) (Table S6).

## 4 Discussion

The present RCT evaluated the impact of different emergence profiles, CONCAVE versus CONVEX, on the stability of the mid‐facial mucosal margin in implant‐supported crowns placed in the aesthetic region. The results predominantly indicate that CONVEX profiles are associated with a significantly higher risk of mid‐facial recession, particularly within the first year following crown placement. In other words, when recessions occur, they tend to develop during the first year after the delivery of the definitive crown, with minimal additional changes thereafter. These findings extend the previously reported 1‐year data from the same cohort, confirming that emergence profile design has its greatest effect early on, with limited influence on the peri‐implant mucosal stability in the longer term.

### 4.1 Mucosal Stability

While recession remained more frequent in the CONVEX group at 3 years (46.7%) compared to the CONCAVE (13.3%) and Control groups (40.0%), these proportions were consistent with those observed at the 1‐year mark. This finding aligns with a previous pilot clinical study, which reported a 13% incidence of recession with CONCAVE emergence profiles (Rompen et al. 2007). The results likely reflect the influence of prosthetic design, particularly convex profiles, on the stability (Esquivel et al. 2024; Gomez‐Meda et al. 2021; Su et al. 2010) and dimensions (Puisys et al. 2023; Rungtanakiat et al. 2024) of the peri‐implant mucosa. A CONVEX emergence profile limits the space available for soft tissue maturation and prevents coronal migration or ‘soft tissue creeping’ during healing (Jonathan Esquivel et al. 2024). Indeed, as shown in the present study, CONVEX restorations were associated with a higher risk of recession. In contrast, a concave profile may facilitate coronal ‘soft tissue creeping’ by providing additional space and adequate ‘running room’ for soft tissue adaptation (Gonzalez‐Martin et al. 2020). A recent pre‐clinical study (Strauss, Park, et al. 2024) demonstrated that a narrow restorative angle, characteristic of CONCAVE profiles, supports the development of a structured, ‘cuff‐like’ soft tissue barrier (Berglundh et al. 1991; Klinge et al. 2006). In contrast, wider angles, resembling CONVEX profiles, were found to impair the formation of a continuous junctional epithelium and compromise its integrity (Strauss, Park, et al. 2024).

The present findings contrast with those of a recent RCT, which compared CONCAVE and linearly divergent CAD/CAM zirconia abutments and found no significant differences within the first year of service (Barwacz et al. 2025). The mentioned study included 54 patients and observed no significant differences between the two prosthetic designs. The discrepancy in our results likely stems from differences in study design. Specifically, our study compared CONVEX to CONCAVE emergence profiles, representing a more pronounced morphological contrast. In contrast, the mentioned RCT compared two relatively similar shapes, CONCAVE and linearly divergent, potentially limiting the ability to detect significant differences (Barwacz et al. 2025).

Another RCT (Koutouzis et al. 2019, 2023), comparing CONVEX and CONCAVE abutments over 3 years, also reported no significant differences between the groups. However, its statistical power was limited due to patient dropouts at 1 and 3 years of follow‐up, reducing the ability to detect changes in the primary outcome (mid‐facial mucosal changes). Additionally, the trial was restricted to maxillary premolars (Koutouzis et al. 2019, 2023), which may account for discrepancies with the present findings. Interestingly, a secondary CBCT analysis of the cohort (Koutouzis and Ali 2021) revealed that CONCAVE designs were associated with thicker peri‐implant mucosa. This observation further supports the current findings that CONCAVE emergence profiles promote mid‐facial mucosal stability through enhanced ‘running room’ and space for soft tissue adaptation (Esquivel et al. 2024; Strauss, Park, et al. 2024).

Interestingly, no statistically significant increase in recession was observed between years one and three. This stability supports the idea that peri‐implant soft tissue changes occur primarily within the first year after restoration, after which the tissues tend to remain stable (Koutouzis et al. 2023). This suggests that tissue maturation mainly occurs during the first year, which may explain the common recommendation to maintain provisional restorations for 3–12 months (Esquivel et al. 2024; Gonzalez‐Martin et al. 2020). However, this rationale is theoretical, as direct histological evidence supporting this concept is lacking.

### 4.2 Aesthetic and Clinical Stability

Despite group differences in soft tissue recession, no significant variation in aesthetic outcomes measured by PES was found across groups at any timepoint. This is not surprising, as PES evaluates five parameters, only one of which reflects the mucosal level. However, when stratifying for this individual parameter, a clear tendency emerged: all but one patient (92%) in the CONCAVE group exhibited a stable mucosal margin with no discrepancy in the level of the facial mucosa, whereas this was observed in only 50% of patients in the CONVEX group. Taken together, this suggests that minor soft tissue displacement may not compromise the perceived aesthetic outcome when restorations are carefully planned in a prosthetically driven position. The absence of a group‐by‐time interaction further implies that soft tissue aesthetics, once established, tend to remain stable.

Clinical parameters, including BOP, did not differ significantly between groups. However, mean and median BOP scores were consistently lower in the CONCAVE group than in the CONVEX group. This observation is in line with findings from a large cross‐sectional study by Pelekos et al. (2023), who reported increased odds of BOP with wider emergence angles in a cohort of 122 participants (Pelekos et al. 2023). The high overall prevalence of mucositis observed across all groups found in the current study may, in part, reflect differences in assessment methodology. BOP can be recorded using varying criteria, for example, punctiform versus profuse bleeding, and is influenced by differences in probing technique (Monje and Salvi 2024). These methodological variations are not standardized across studies, which may contribute to the overestimation of disease prevalence. Furthermore, BOP around implants is not solely indicative of inflammation; it can also be influenced by factors such as probing force and technique. BOP may be present even in clinically healthy peri‐implant tissues (Monje and Salvi 2024). For instance, increasing probing force from 0.15 to 0.25 N has been shown to significantly increase BOP rates (Gerber et al. 2009), highlighting the sensitivity of this measure to minor procedural variations. Thus, reliance on a dichotomous BOP classification may compromise diagnostic accuracy and partly account for the high prevalence of mucositis reported in this study.

### 4.3 Dimensional and Radiographic Outcomes

Buccal soft tissue contours, assessed using digital profilometry, confirmed the early remodelling phase observed at 1 year, with negligible further change thereafter. While ROI‐3 revealed group‐level differences, these did not translate into clinically meaningful soft tissue loss. Marginal bone levels remained stable over time, and no significant differences were observed between groups at any follow‐up interval. The observed values confirm that marginal bone remodelling predominantly occurs within the first year and thereafter enters a steady state (Albrektsson et al. 2022).

### 4.4 Clinical Relevance

These findings reinforce the need for early control of soft tissue contours during the provisional phase (Esquivel et al. 2024). The emergence profile established at the time of provisionalization appears to shape the peri‐implant environment durably. As outlined by previous anecdotal evidence (Esquivel et al. 2024; Gomez‐Meda et al. 2021; Su et al. 2010) digital protocols for replicating the provisional emergence profile offer a predictable method for transferring these contours to the definitive restoration (Lanis et al. 2024; Pedrinaci et al. 2024).

It should be noted that a convex shape may be necessary in certain cases to prosthetically compensate for excessive palatal implant placement or angulation (Chu et al. 2019; Steigmann et al. 2014) to address minor soft tissue deficiencies (e.g., ridge concavity) or to promote apical relocation of the mucosal margin (Gonzalez‐Martin et al. 2020). However, the latter should be applied with caution to minimize the risk of further recessions. Anecdotal evidence (Chu et al. 2019; Esquivel et al. 2021; Su et al. 2010) suggests that a prosthetic design featuring a concave subcritical contour combined with a convex critical contour may allow for controlled induction of soft tissue recession.

### 4.5 Limitations

The present study has limitations. Recession was measured by the apical shift of the mid‐facial mucosa using a periodontal probe. As values often fell within the probe's error margin (~1 mm) (Badersten et al. 1984; Grossi et al. 1996), random measurement error cannot be ruled out. Moreover, a defined threshold, range or precise angle for what was considered concave or convex was not measured numerically. Thus, the degree of customization chairside could not be fully standardized due to the need for individual customization based on the patient's soft tissue anatomy, implant position and tooth location (Chu et al. 2019). Furthermore, no patient‐reported outcomes were evaluated (Jung et al. 2025; Thoma and Strauss 2022; Weinfurt and Reeve 2022). Finally, whether these findings are applicable for the posterior remains unclear and should be examined in future studies.

## 5 Conclusion

The emergence profile design significantly influences soft tissue stability around anterior implants, primarily within the first year following crown insertion. A CONCAVE profile showed superior soft tissue stability, particularly in preventing mid‐facial recessions. Therefore, when clinically feasible, a CONCAVE design is preferable in the aesthetic zone.

## Author Contributions

All authors made substantial contributions to this study. D.S.T. and R.E.J. contributed to the conception and design of the study. F.J.S., J.E., M.S. and N.N. contributed to the clinical phases of the study and collected the data. F.J.S. performed the statistical analyses and interpreted the data and. F.J.S. and J.E. drafted the manuscript and M.S., R.E.J., N.N. and D.S.T. critically reviewed and revised the manuscript.

## Conflicts of Interest

The authors declare no conflicts of interest.

## Supporting information


**Data S1:** jcpe70018‐sup‐0001‐supinfo.docx.

## Data Availability

The data that support the findings of this study are available on request from the corresponding author. The data are not publicly available due to privacy or ethical restrictions.
