# Supplementary material for: Convex Versus Concave Emergence Profile of Implant‐Supported Crowns in the Aesthetic Zone: 3‐Year Results of a Randomized Controlled Trial
Source: J Clin Periodontol. 2025 Aug 20;52(11):1605–15. doi: 10.1111/jcpe.70018 (PMC12531365; doi:10.1111/jcpe.70018)
Supplement: Supplementary file 1 — Data S1: jcpe70018‐sup‐0001‐supinfo.docx. [file JCPE-52-1605-s001.docx]

**Supplement tables, figures and appendix**

**Tables**

**Supplement Table 1.** Participants baseline characteristics.

|  | **Group Convex** | **Group Concave** | **Group Control** | **All patients** |  |
| --- | --- | --- | --- | --- | --- |
| N (%) | 15 (32%) | 16 (34%) | 16 (34%) | 47 (100%) |  |
| Age, mean | 59.2 | 60.9 | 60.8 | 60.3 |  |
| Gender |  |  |  |  |  |
| Female | 4 (27%) | 10 (63%) | 6 (38%) | 20 (43%) |  |
| Male | 11 (73%) | 6 (37%) | 10 (62%) | 27 (57%) |  |
| Location, N (%) |  |  |  |  |  |
| Incisors | 4 (9%) | 4 (9%) | 2 (4%) | 10 (21%) |  |
| Canines | 1 (2%) | 4 (9%) | 1 (2%) | 6 (13%) |  |
| Premolars | 10 (21%) | 8 (17%) | 13 (28%) | 31 (66%) |  |
| Biotype, N (%) |  |  |  |  |  |
| Thick | | 14 (30%) | 13 (28%) | 13 (28%) | 40 (86%) |
| Thin | | 1 (2%) | 3 (6%) | 3 (6%) | 7 (14%) |

Data are presented as N (%) or mean (SD).

**Supplement Table 2.** Multivariable logistic regression analysis for predicting the presence of recessions (yes/no) adjusted for treatment, soft tissue thickness and keratinized tissue width (ITT analysis applying Last Observation Carried Forward method for handling missing data, n=45)

|  | 3 years follow-up | | |
| --- | --- | --- | --- |
|  | OR | 95% CI | p-value |
| Treatment |  |  |  |
| CONCAVE  (reference) | 1 |  |  |
| CONVEX | 4.8 | 0.8 – 27.4 | 0.072 |
| CONTROL | 1.9 | 0.3 – 10.5 | 0.423 |
| Soft tissue thickness (mm) | 1.2 | 0.8 – 1.9 | 0.217 |
| Keratinized tissue width (mm) | 0.7 | 0.4 – 1.2 | 0.281 |

Abbreviation: OR, Odds-Ratio; CI, confidence interval

**Supplement Table 3.** Multivariable logistic regression analysis for predicting the presence of recessions (yes/no) adjusted for treatment (n=42)

|  | 3 years follow-up | | |
| --- | --- | --- | --- |
|  | OR | 95% CI | p-value |
| Treatment |  |  |  |
| CONCAVE  (reference) | 1 |  |  |
| CONVEX | 1.7 | -0.1 – 3.5 | 0.069 |
| CONTROL | 1.2 | -0.5 – 3.1 | 0.163 |

Abbreviation: OR, Odds-Ratio; CI, confidence interval

**Supplement Table 4.** Multivariable logistic regression analysis for predicting the presence of recessions (yes/no) adjusted for treatment and keratinized tissue width (n=42)

|  | 3 years follow-up | | |
| --- | --- | --- | --- |
|  | OR | 95% CI | p-value |
| Treatment |  |  |  |
| CONCAVE  (reference) | 1 |  |  |
| CONVEX | 5.4 | 0.8 – 34.1 | 0.073 |
| CONTROL | 3.6 | 0.5 – 22.6 | 0.172 |
| Keratinized tissue width (mm) | 0.95 | 0.5 – 1.5 | 0.862 |

Abbreviation: OR, Odds-Ratio; CI, confidence interval

**Supplement Table 5.** Multivariable logistic regression analysis for predicting the presence of recessions (yes/no) adjusted for treatment and soft tissue thickness (n=42)

|  | 1 year to 3 years follow-up | | |
| --- | --- | --- | --- |
|  | OR | 95% CI | p-value |
| Treatment |  |  |  |
| CONCAVE  (reference) | 1 |  |  |
| CONVEX | 7.4 | 1.0 – 52.8 | 0.046 |
| CONTROL | 3.3 | 0.5 – 21.6 | 0.196 |
| Soft tissue thickness (mm) | 1.2 | 0.8 – 1.8 | 0.285 |

Abbreviation: OR, Odds-Ratio; CI, confidence interval

**Supplement Table 6.** Marginal bone levels within each group across and timepoints

| Mean marginal bone level (mm) | | | | | | | | | |
| --- | --- | --- | --- | --- | --- | --- | --- | --- | --- |
| Group | **CONVEX** | | **CONCAVE** | | **CONTROL** | |  | | |
| Timepoint | Mean (SD) | Median (Q1, Q3) | Mean (SD) | Median (Q1, Q3) | Mean (SD) | Median (Q1, Q3) | p-value  (shape effect) | p-value  (time effect) | p-value  (angle#time interaction) |
| Baseline | 0.52 (0.38) | 0.37 (0.22, 0.73) | 0.51 (0.20) | 0.54 (0.32, 0.70) | 0.47 (0.26) | 0.40 (0. 25, 0.71) | 0.961 | 0.805 | 0.301 |
| 1-year follow-up | 0.42 (0.36) | 0.34 (0.13, 0.72) | 0.28 (0.27) | 0.27 (0.08, 0.50) | 0.32 (0.24) | 0.32 (0.14, 0.50) |  |  |  |
| 3-year follow-up | 0.45 (0.98) | 0.25 (-0.00, 0.62) | -0.02 (0.69) | -0.00 (-0.25, 0.29) | 0.05 (0.64) | 0.21 (0.04, 0.39) |  |  |  |

Note: Differences were tested using a linear mixed effect model with treatment (shape), time and their interaction P-values are indicated. Wald's Chi-squared statistic was used to conclude about main effects and interactions between treatment and time. Abbreviations: SD, standard deviation; Q1, first quartile; Q3, third quartile.

**Supplement Figures**


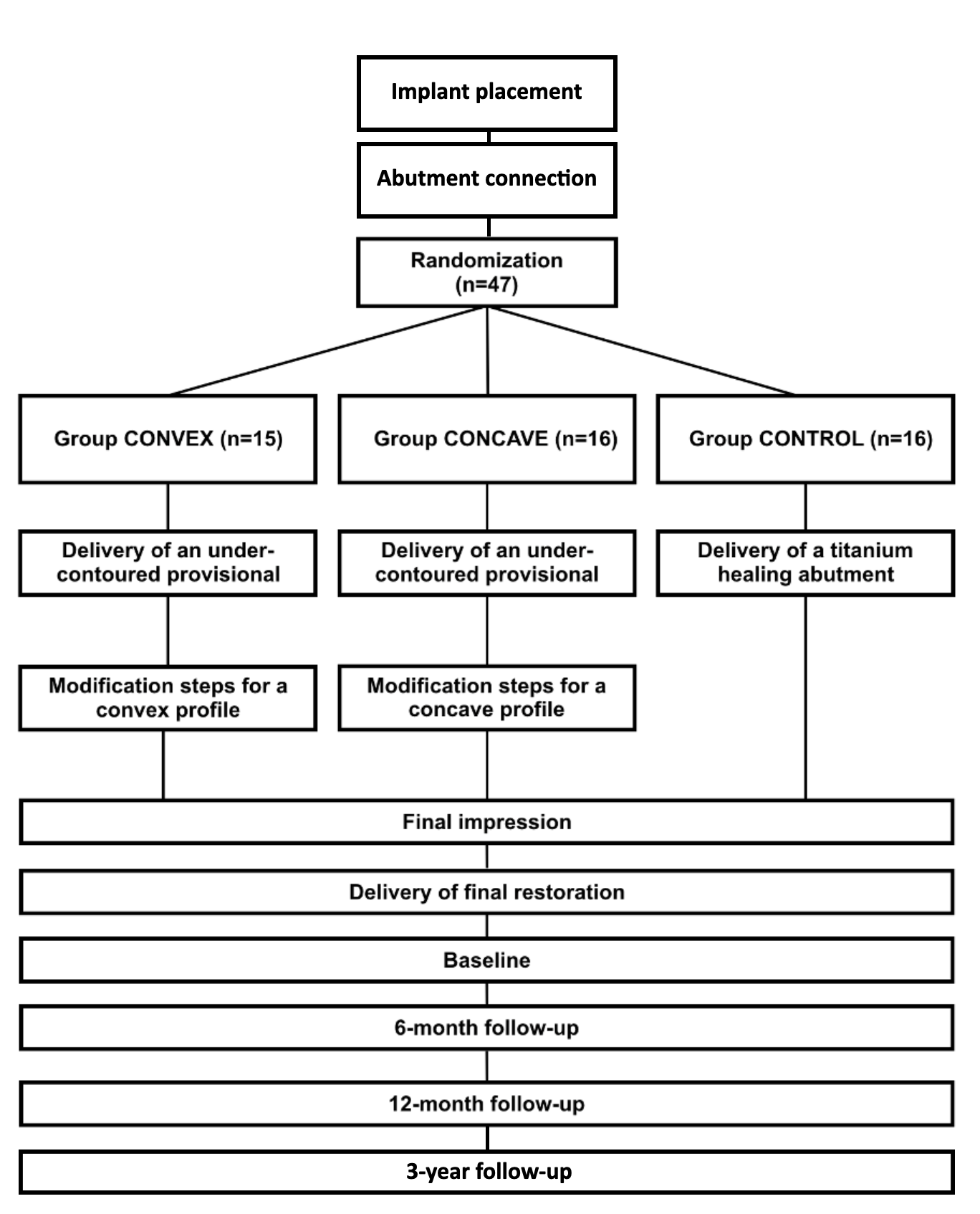


**Supplement Figure 1.** Study timeline (updated from Siegenthaler et al., 2022)

**
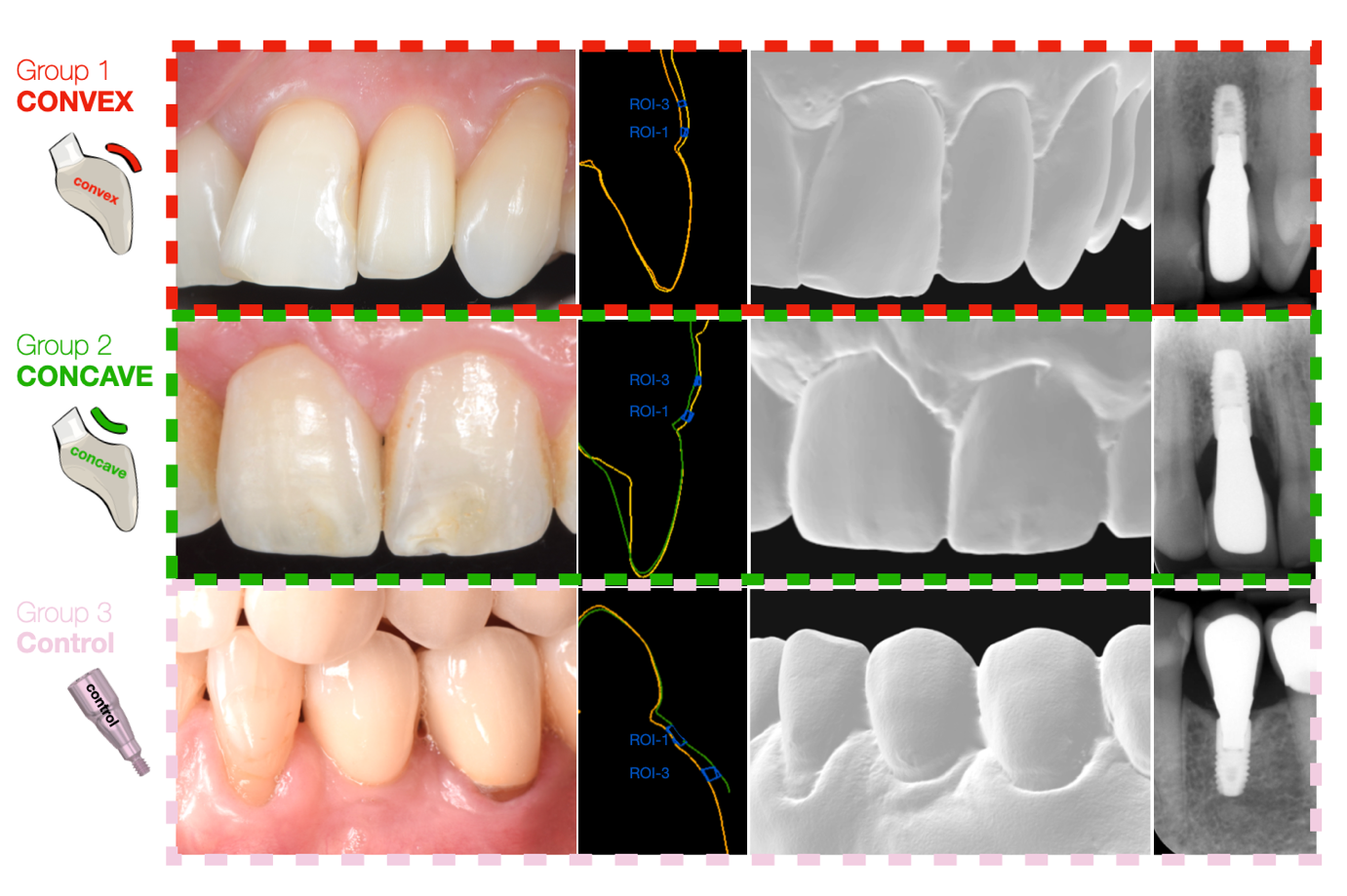
**

**Supplement Figure 2.** Profilometric analysis across treatment groups at two regions of interest (ROIs): 1 mm (ROI-1) and 3 mm (ROI-2) apical to the midfacial mucosal margin.


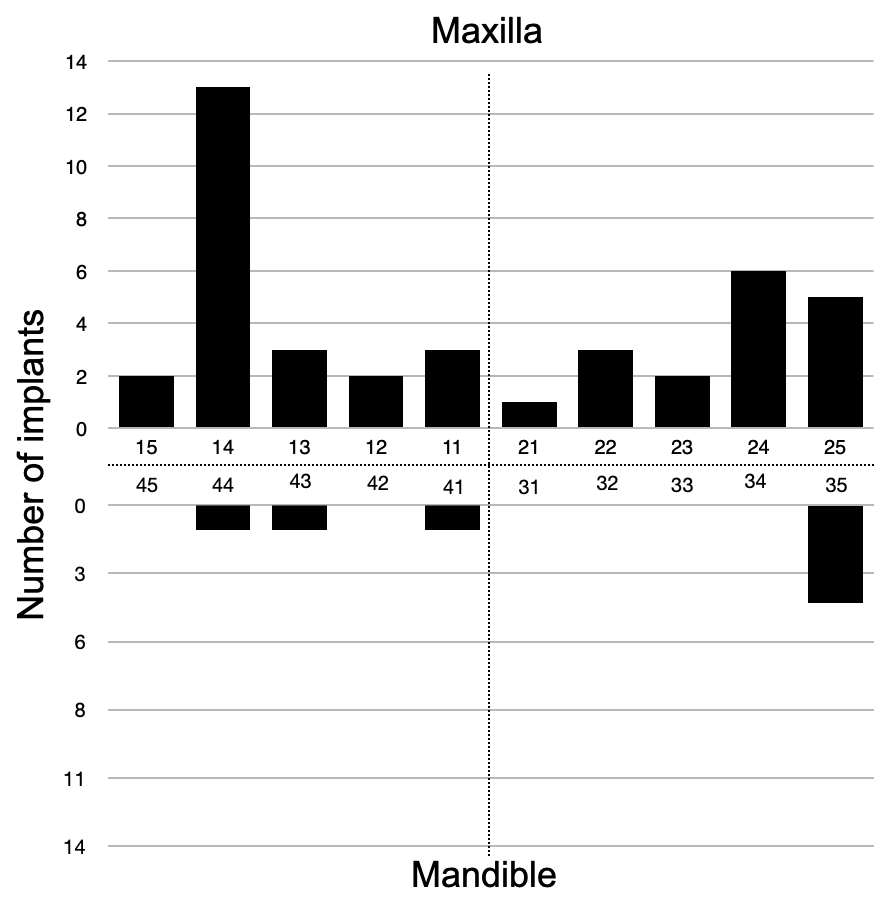


**Supplement Figure 3.** Number of implants and their distribution.
